# Supplementary material for: Age is an intrinsic driver of inflammatory responses to malaria
Source: Nat Commun. 2025 Sep 30;16:8665. doi: 10.1038/s41467-025-63638-1 (PMC12485045; doi:10.1038/s41467-025-63638-1)
Supplement: Supplementary file 1 — Supplementary Information [file 41467_2025_63638_MOESM1_ESM.pdf]

Supplementary Tables and Figures

Supplementary Table S1: Demographics of patients analyzed for plasma cytokines

**Demographics**	**Total cohort**	**District hospital#**	**Referral hospital^**
**Number**	97	79	18
**Age,** years, median [IQR], range	21, [15-45], 2-72	21 [14-40.5], 2-72	24.5 [18.25-45.75], 14-66
**Male sex**, n (%)	78 (80%)	61 (77%)	17 (94%)
**Parasites/ µL,** median (IQR)	18 525 (2 522-63 612)	9 924 (2030-32 839)	233 409 (142 012-425 315)
**Severe malaria$,** n (%)	24 (24.7%)	6 (7.6%)	18 (100%)

Samples from District Hospital enrollments #ref24, Grigg MJ, et al. Age-Related Clinical Spectrum of Plasmodium knowlesi Malaria and Predictors of Severity. *Clinical Infectious Diseases*. 2018;67(3):350–359. All available samples from this cohort were tested

^ Samples from referral hospital #ref25. Barber BE, et al. A prospective comparative study of knowlesi, falciparum, and vivax malaria in Sabah, Malaysia: high proportion with severe disease from Plasmodium knowlesi and Plasmodium vivax but no mortality with early referral and artesunate therapy. *Clinical Infectious Diseases*. 2013;56(3):383–397. A subset of participants were tested selected based on plasma volume availability.

$ Severe malaria was defined using World Health Organization 2014 research criteria.

Supplementary Table S2: Malaria clinical cohort characteristics district hospital cohort

**Patient characteristic**	***Children*** *≤12 yrs*	***Adolescents/adults*** *>12 yrs*	**P-value** *two-sided*
**Number** (% total)	31 (32.3)	65 (67.7)	-
Age, years			
Median (IQR)	7 (3-10)	24 (16-47)	-
Range	1-12	1-12	
Male sex, n (%)	21 (67.7)	48 (73.8)	0.534
Previous malaria (self-reported), n (%)	3 (9.7)	8 (12.7)	0.705
Days of fever, median (IQR)	4 (3-5)	4 (3-6)	0.237
Symptoms on enrolment, n (%)			
Rigors	14 (45.2)	49 (76.6)	**0.004**
Headache	21 (67.7)	56 (86.2)	**0.034**
Vomiting	11 (35.5)	28 (43.1)	0.479
Abdominal pain	9 (29.0)	18 (27.7)	0.891
Diarrhoea	4 (12.9)	7 (10.8)	0.759
Cough	12 (38.7)	22 (33.8)	0.712
Shortness of breath	4 (12.9)	10 (15.4)	0.741
Myalgia	7 (22.6)	35 (53.8)	**0.004**
Arthralgia	7 (22.6)	34 (52.3)	**0.006**
Examination findings on enrolment			
Oxygen saturation, %, median (IQR)	100 (99-100)	99 (98-100)	**0.010**
Parasite count, parasites/µL, median (IQR)	7392 (1462-36546)	9924 (2522-22860)	0.934

Supplementary Table S3: Malaria naive cohort information

	**Children <12 years**	**Adults >12 years**	**P**
Total, n (%)	13, (50%)	13, (50%)	-
Sex, male, n (%)	8, (61%)	6, (46%)	ns[#]
CMV, positive, n (%)	8, (61%)	9, (69%)	ns[#]
Age years, median [IQR]	8 [3-12]	42 [29-46]	p < 0.0001^
Allergies[$]	Dairy (n=1), dust mite (n=3), egg (n=2), grass pollen (n=1), nuts (n=2), beta lactam (n=1)	Dust mite (n=2), nuts (n=2), penicillin (n=1), bee venom (n=1), crustacean (n=1), salmon (n=1), non-allergy rhinitis (n=1)	-
Average time since last reaction[$]	4 months- over 2 years	2 months- >10 years	-

Chai square
^ Mann-Whitney U test
$ data was available for n=10 children and n=9 adults
P are two sided with no adjustments for multiple comparisons

Supplementary Table S4: Antibody panel for ex vivo phenotyping

Antigen	Fluorochrome	Clone	Manufacturer	Cat #	Dilution
CXCR3	BV421	1C6	BD Biosciences	562558	1/50
CD86	BV480	2331	BD Biosciences	566131	1/100
CD14	BV510	M5E2	Biolegend	301842	1/50
CD127	BV570	A019D5	Biolegend	351308	1/100
CCR6	BV650	11A9	BD Biosciences	563922	1/100
CXCR5	BV711	J252D4	Biolegend	356934	1/50
HLA-DR	BV785	L243	Biolegend	307642	1/50
CD45RA	BB515	HI100	BD Biosciences	564552	1/1000
CD3	FITC	SK7	Biolegend	344804	1/10
CD4	PerCPCy5.5	OKT4	Biolegend	317428	1/400
CD19	PE	HIB19	Biolegend	302208	1/10
CD56	PE-dazzle	HCD56	Biolegend	318347	1/100
PD-1	PE-CY7	EH12.1	BD Biosciences	561272	1/100
Vδ2	APC	B6	Biolegend	331418	1/500
FoxP3	AF647	206D	Biolegend	320114	1/25
CD16	AF700	3G8	Biolegend	302026	1/2500
ICOS	APC-Cy7	C398.4A	Biolegend	301820	1/100
Viability	NIR		Invitrogen	L34975	1/1500

Supplementary Table S5: Antibody panel for innate cell parasite stimulation

Antigen	Fluorochrome	Clone	Manufacturer	Cat #	Dilution
IL-12	BV421	C8.6	BD Biosciences	565023	1/33
CD86	BV480	2331	BD Biosciences	566131	1/100
HLADR	BV570	L243	Biolegend	307637	1/100
IFNγ	BV605	B27	BD Biosciences	562974	1/25
CD27	BV650	O325	Biolegend	302827	1/400
CD45RA	BV711	H100	Biolegend	304137	1/1000
TNF	BV750	Mab11	BD Biosciences	566359	1/100
CD3	AF352	UCHT1	Invitrogen	58003842	1/50
IL-1β	FITC	CRM56	Invitrogen	11-7018-42	1/100
CD14	PerCPCy5.5	M5E2	Biolegend	301823	1/400
IL-10	PE	JES3-9D7	BD Biosciences	559337	1/10
CD56	PE-dazzle	HCD56	Biolegend	318347	1/50
IL-6	PE-Cy7	MQ2-13A5	Biolegend	501119	1/100
Granzyme B	APC	QA16A02	Biolegend	372203	1/1000
MCP-1	AF647	5D3-F7	BD Biosciences	563496	1/150
CD16	AF700	3G8	Biolegend	302026	1/2500
Vδ2	APC-FIRE	B6	Biolegend	331419	1/500
Viabilty	NIR		Invitrogen	L34975	1/1500

Supplementary Table S6: Antibody panel for CD4 T cell parasite stimulation

Antigen	Fluorochrome	Clone	Manufacturer	Cat #	Dilution
FOXP3	BV421	206D	Biolegend	320124	1/25
CXCR3	PAC Blue	G025H7	Biolegend	353723	1/50
CD127	BV570	A019D5	Biolegend	351308	1/100
CCR4	BV605	L291H4	Biolegend	359417	1/100
CCR6	BV650	11A9	BD Biosciences	563922	1/100
CXCR5	BV711	J252D4	Biolegend	356934	1/50
CCR7	BV786	3D12	BD Biosciences	563710	1/50
CD45RA	BB515	HI100	BD Biosciences	564552	1/1000
Ki67	FITC	B56	BD Biosciences	556026	1/100
CD3	AF352	UCHT1	Invitrogen	58003842	1/50
CD4	PerCPCy5.5	OKT4	Biolegend	317428	1/100
PD-1	PE-Cy7	EH12.1	BD Biosciences	561272	1/100
TNFR2	AF647	hTNFR-M1	BD Biosciences	562909	1/50
CD25	AF700	2A3	BD Biosciences	565106	1/250
ICOS	APC-Cy7	C398.4A	Biolegend	301820	1/100
Viabilty	NIR		Invitrogen	L34975	1/1500

Supplementary Table S7: Antibody panel for FACS sorting

Antigen	Fluorochrome	Clone	Manufacturer	Cat #	Dilution
Viability	Sytox Blue	-	Invitrogen	S11348	1/200
CD56	BV510	HCD56	Biolegend	318340	1/50
CD19	BV605	SJ25C1	BD Biosciences	562653	1/50
CD3	BV650	OKT3	Biolegend	317324	1/50
HLA-DR	BV785	L243	BioLegend	307642	1/50
CD14	PE	M5E2	BD	561707	1/50
CD64	PE-DAZZLE 594	10.1	Biolegend	305032	1/100
CD4	PerCP-Cy5.5	OKT4	Biolegend	317428	1/25
Vδ2	APC	B6	BioLegend	331418	1/100

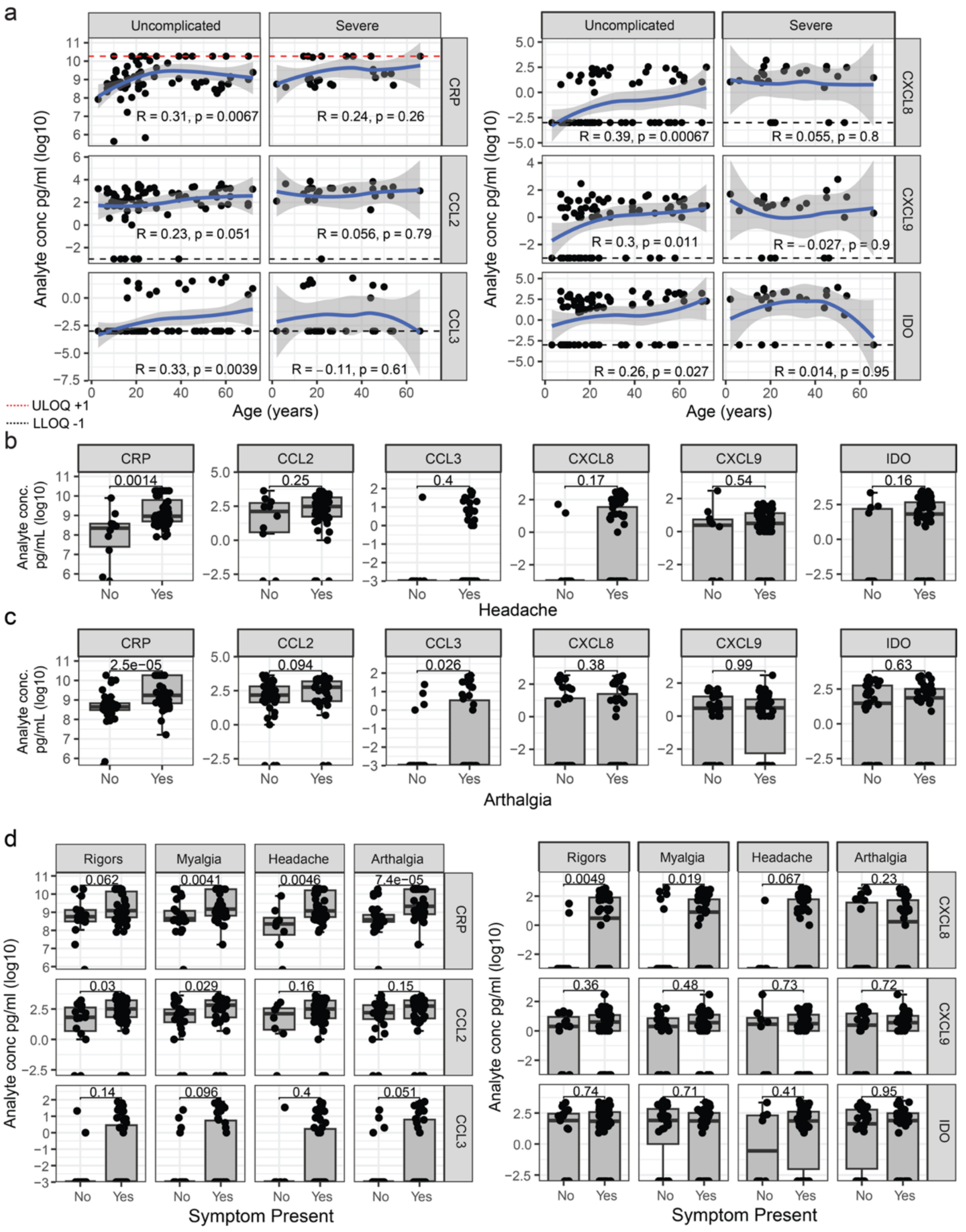

Supplementary Figure 1. Associations between age, cytokines and symptoms. Thirteen analytes were measured in plasma from 97 individuals with acute malaria (male 78 (80%), age 21 [15-45] median [IQR])). **(a)** Age-associated inflammatory analytes stratified by malaria severity. Dashed Horizontal lines represent upper limit of quantification plus one (red: ULOQ +1) and lower limit of quantification minus one (black: LLOQ -1). Analyte concentration for patients with presence of symptoms **(b)** headache or **(c)** arthralgia (joint pain). **(d)** Analyte concentration for patients with presence of symptoms only in adults >12 years of age. Dots represent individuals. Tukey boxplots used for discrete comparisons with Mann-Whitney U test. All p are two-sided, with no adjustments for multiple comparisons. Solid lines are LOESS fit curves with error bands of 95% confidence interval. Spearman's *Rho* and p are indicated.

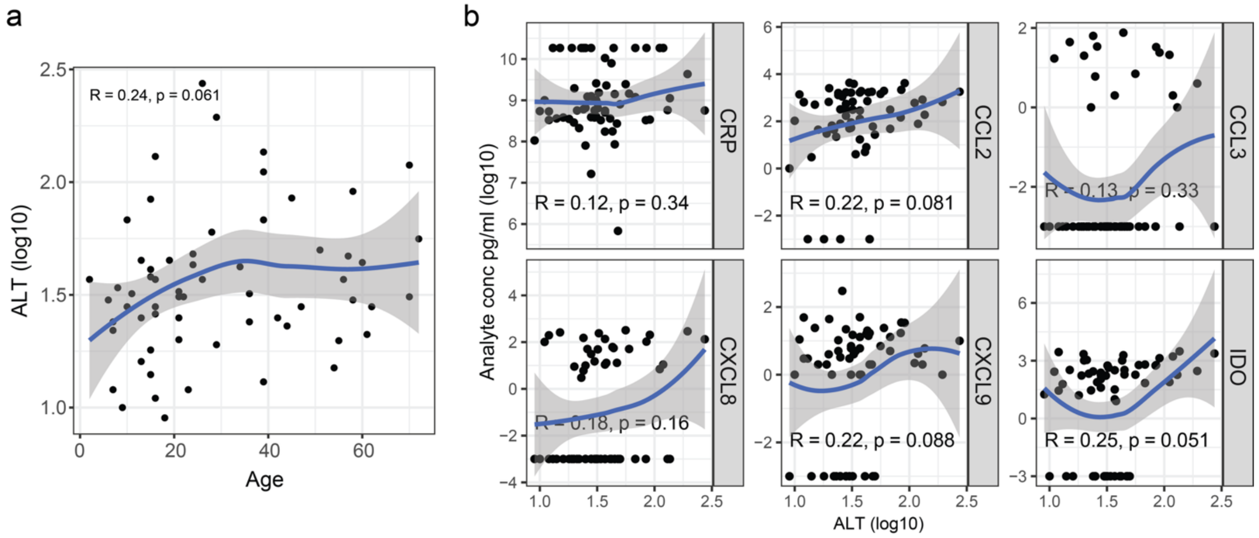

Supplementary Figure 2. ALT associations with age and cytokines. Thirteen analytes were measured in plasma from 97 individuals with acute malaria (male 78 (80%), age 21 [15-45] median [IQR])). **(a)** Correlation plot between ALT and age. **(b)** Individual correlation plots of analytes with ALT. Dots represent individuals. Mann-Whitney U test was used for comparisons between no symptoms and yes symptom. Solid lines are LOESS fit curves with error bands of 95% confidence interval. Spearman's *Rho* and p are indicated. ALT = alanine aminotransferase.

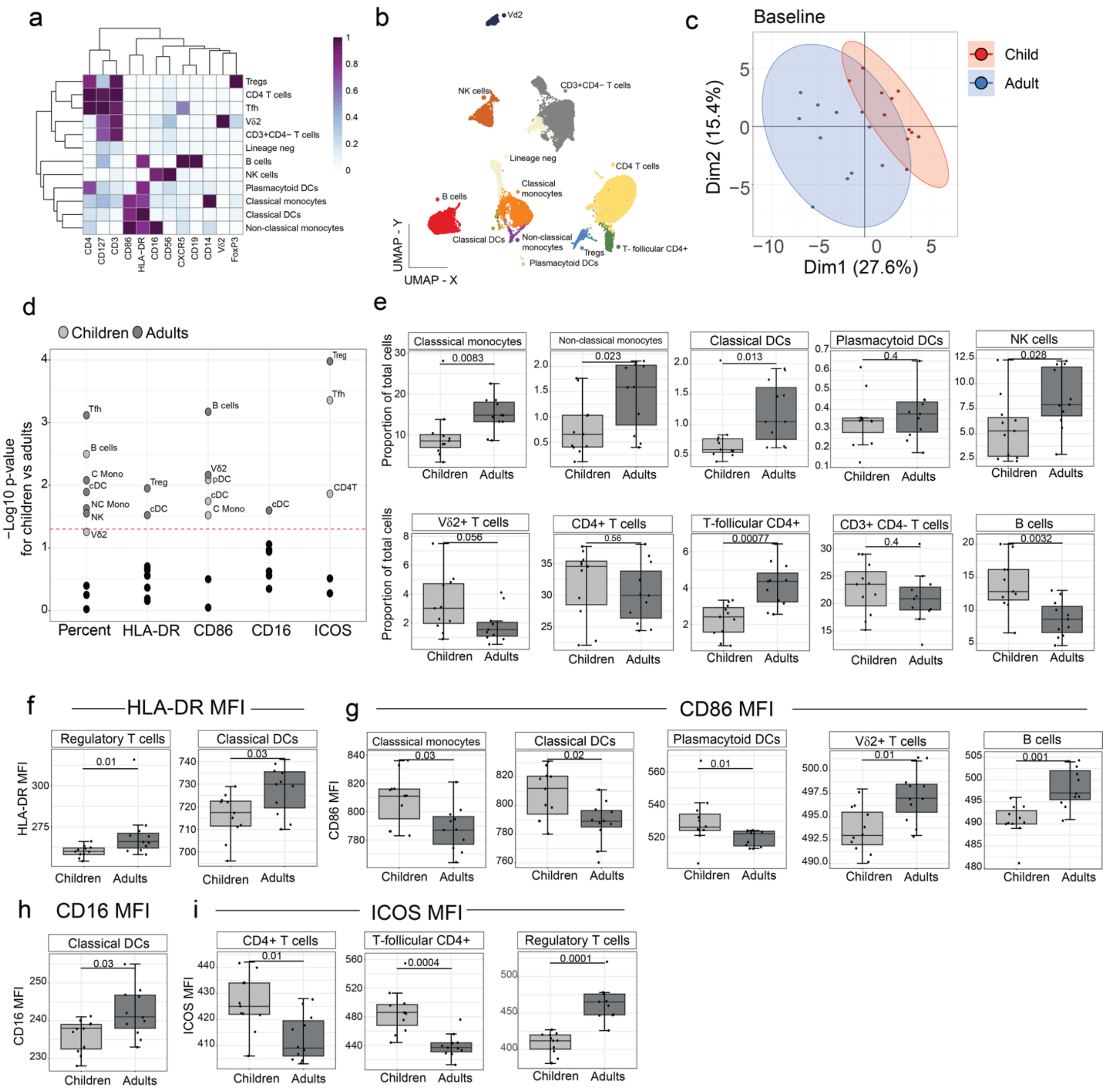

Supplementary Figure 3. Frequency and phenotypes of malaria responsive immune populations in naïve children and adults. High-level cell lineages were identified by cell surface staining of malaria naive adult and children PBMCs using spectral flow cytometry. **(a)** Heatmap of median fluorescence expression (MFI) of protein markers within annotated PBMCs. Heatmap colour is normalized marker expression. **(b)** Uniform manifold approximation and projection (UMAP) of identified immune cell subsets in children (*n*=11) and adults (*n*=11). 11 distinct cell clusters (excluding lineage negative) were identified. **(c)** PCA of cell subset proportions and activation marker expression between children (red) and adults (blue). **(d)** Comparison of age specific differences between children and adults. The –log10 p-value calculated from Mann-Whitney test is indicated for the proportion (% of live PBMCs), HLA-DR MFI, CD86 MFI, CD16 MFI and ICOS MFI. **(e)** Immune cell type frequencies (as % of total cells), and significantly different **(f)** HLA-DR MFI, **(g)** CD86 MFI, **(h)** CD16 MFI, and **(i)** ICOS between children and adults. P values are Mann-Whitney U test. Centre line representing the median, box limits indicating the upper and lower quartiles, whiskers extending to the upper and lower limits. All p are two sided, with no adjustment for multiple comparisons. For all panels data is from children *n*=11 and adults *n*=11. MFI = median fluorescence intensity.

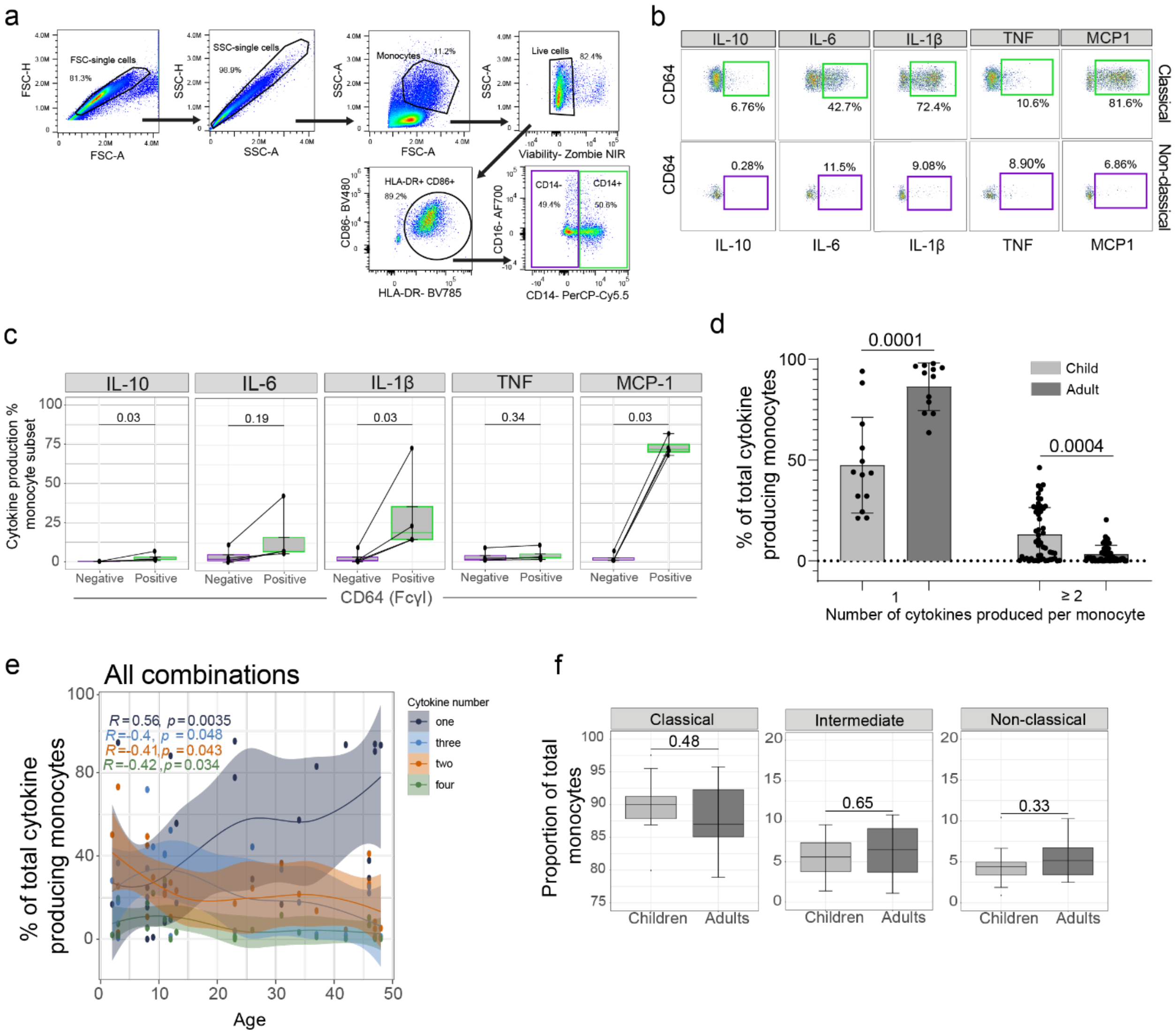

Supplementary Figure 4*. *Functional cytokine response of classical monocytes. (**a**) Flow cytometry gating example for identifying classical and non-classical monocytes after stimulation. Total monocytes were identified as HLADR$^+$ and CD86$^+$, classical monocytes were identified as CD14$^+$ (green box) and non-classical monocytes (purple box) were identified as CD14$^-$. To confirm the different functional potential of our identified monocyte subsets, we analysed their cytokine production in healthy adults (**b**) example flow cytometry plots (**c**) quantification of cytokine response from classical (green) and non-classical monocytes (purple). **(d)** Proportion of cytokine producing classical monocytes producing 1 or ≥ 2 cytokines. **(e)** Correlation between number of cytokines produced by monocytes and age. One cytokine is orange, two cytokines are green, three cytokines are purple, and four cytokines are yellow. Solid lines are LOESS fit curves with error bands of 95% confidence interval. Spearman's correlation and p-values are indicated. **(f)** Proportion of classical, intermediate and non-classical monocytes in children and adults. Lines represent paired observations, and negative and positive comparisons are Wilcox rank paired test. Centre line representing the median, box limits indicating the upper and lower quartiles, whiskers extending to the upper and lower limits. All p are two sided, with no adjustment for multiple comparisons. For all panels data is from children *n*=13 and adults *n*=12

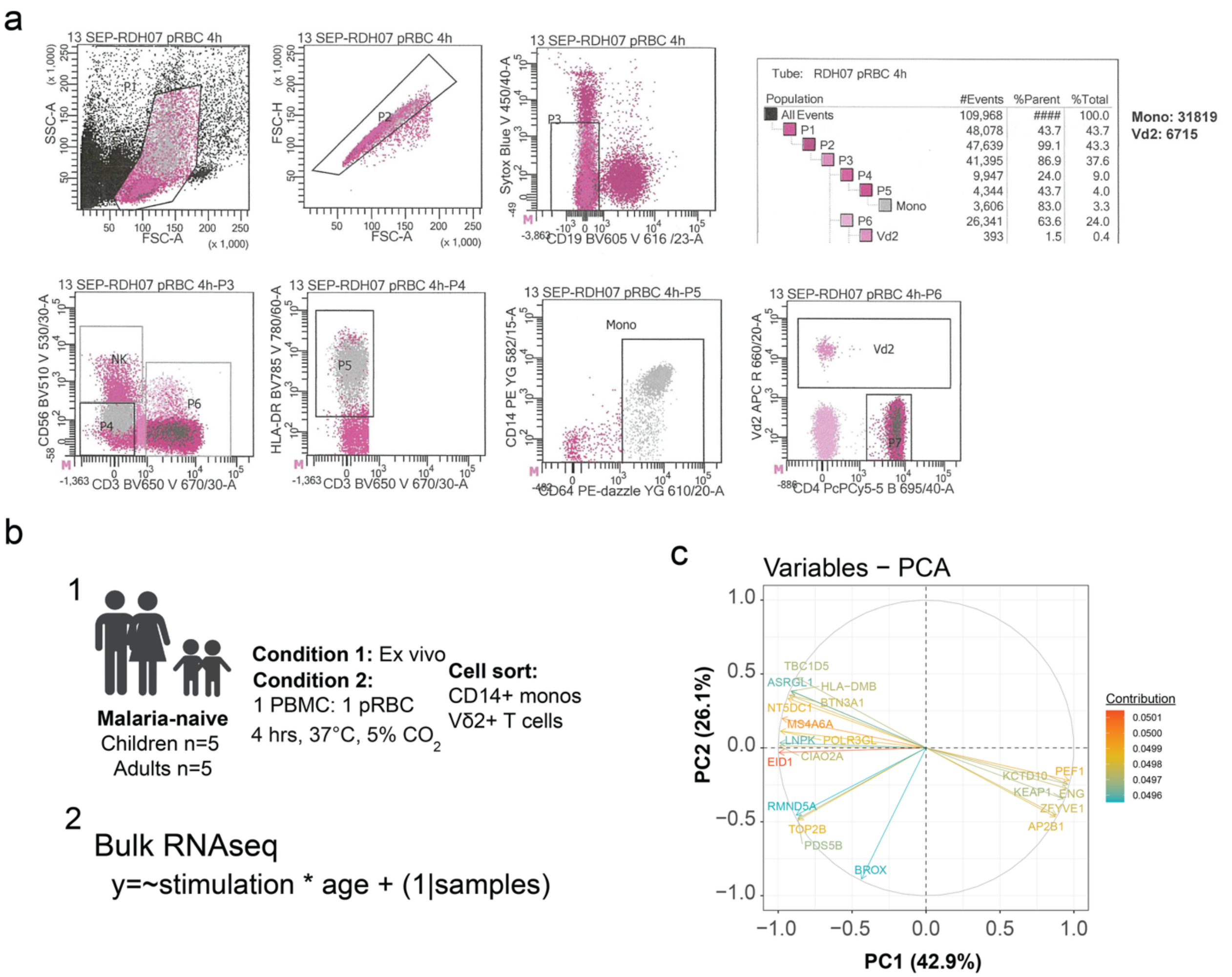

Supplementary Figure 5. Classical monocyte bulk-RNA sequencing. **(a)**. Cell sort gating example, we identified classical monocytes as HLA-DR^{+} CD14^{+} and Vδ2^{+} γδ T cells as CD3^{+} Vδ2^{+}. **(b)** Schematic of experimental (1) and analytical approach (2). CD14^{+} classical monocytes were sorted ex vivo or following parasite stimulation from children (*n*=5) and adults (*n*=5). Created in BioRender. Boyle, M. (2025) https://BioRender.com/n7674u1. **(c)** Specific DEGs driving the variance for PCA1 and PCA2 in monocytes. PCA= Principle Components Analysis.

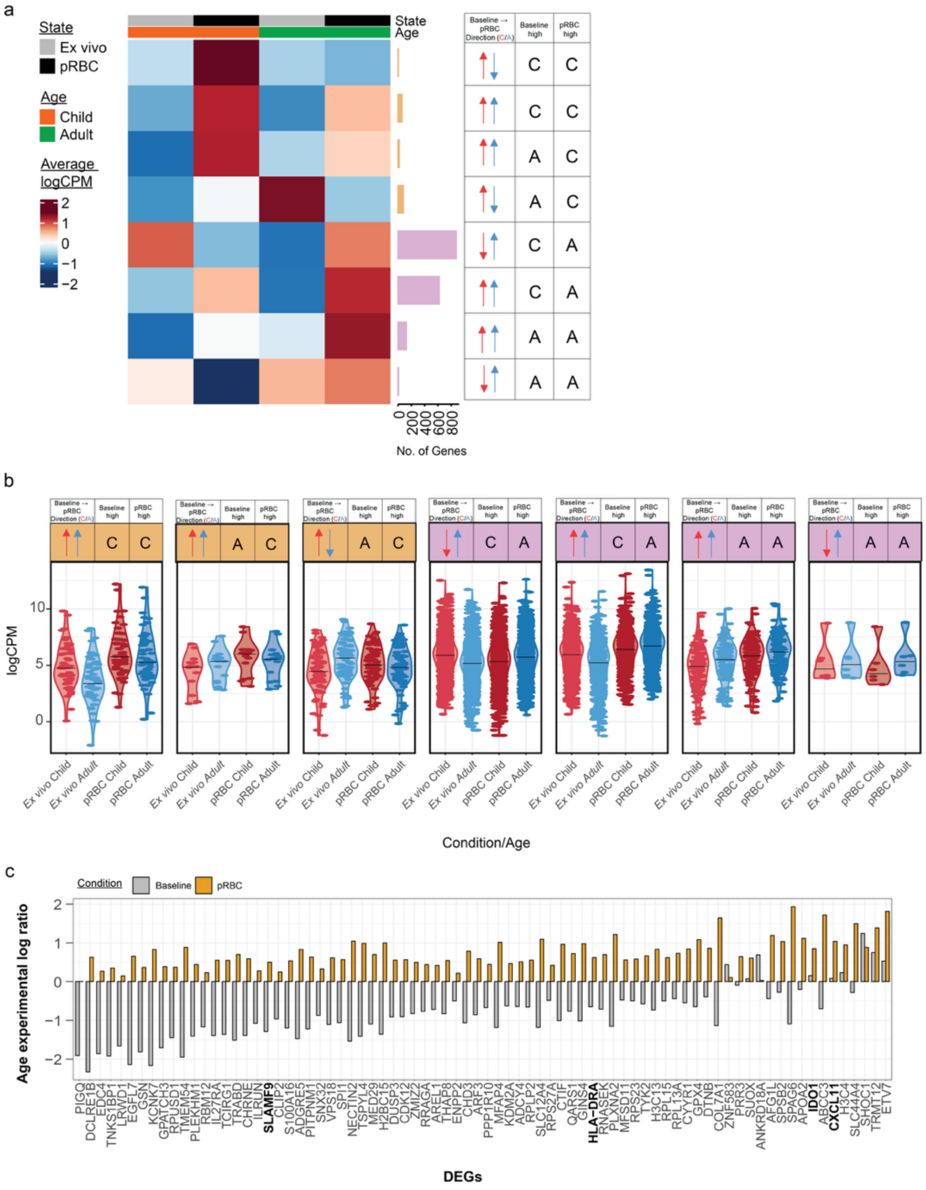

Supplementary Figure 6. DEG grouping for IPA analysis. DEGs grouped based on their direction of change and relative expression levels maximum in children or adults at *Ex vivo* (baseline) and after pRBC stimulation. **(a)** Heatmap depicts the average log2 counts per million (logCPM) of DEGs with number of DEGs, from groups described in Figure 3d, in each age and stimulation conditions. (**b**) Violin plot depicts the logCPM of DEGs, from groups described in Figure 3d, in each age and stimulation condition. One child group was omitted due to only containing one DEG. Purple bars indicate genes that increased expression levels after stimulation and were higher in adults. Orange bars indicate genes that increased expression levels after stimulation and were higher in children. **(c)** DEGs from classical monocytes with a qvalue (age & stim) < 0.001 (82 genes). A positive age experimental log ratio for a DEG indicates it was higher in adults before or after stimulation and a negative age experimental log ratio for a DEG indicates it was higher in children before or after stimulation. Grey bars are *ex vivo* (baseline) values and orange bars are pRBC stimulated values. For example, CXCL11 (bold) were higher in adults before and after stim (positive age experimental log ratio values). For all panels data is from children *n*=5 and adults *n*=5. pRBC = parasitised red blood cells.

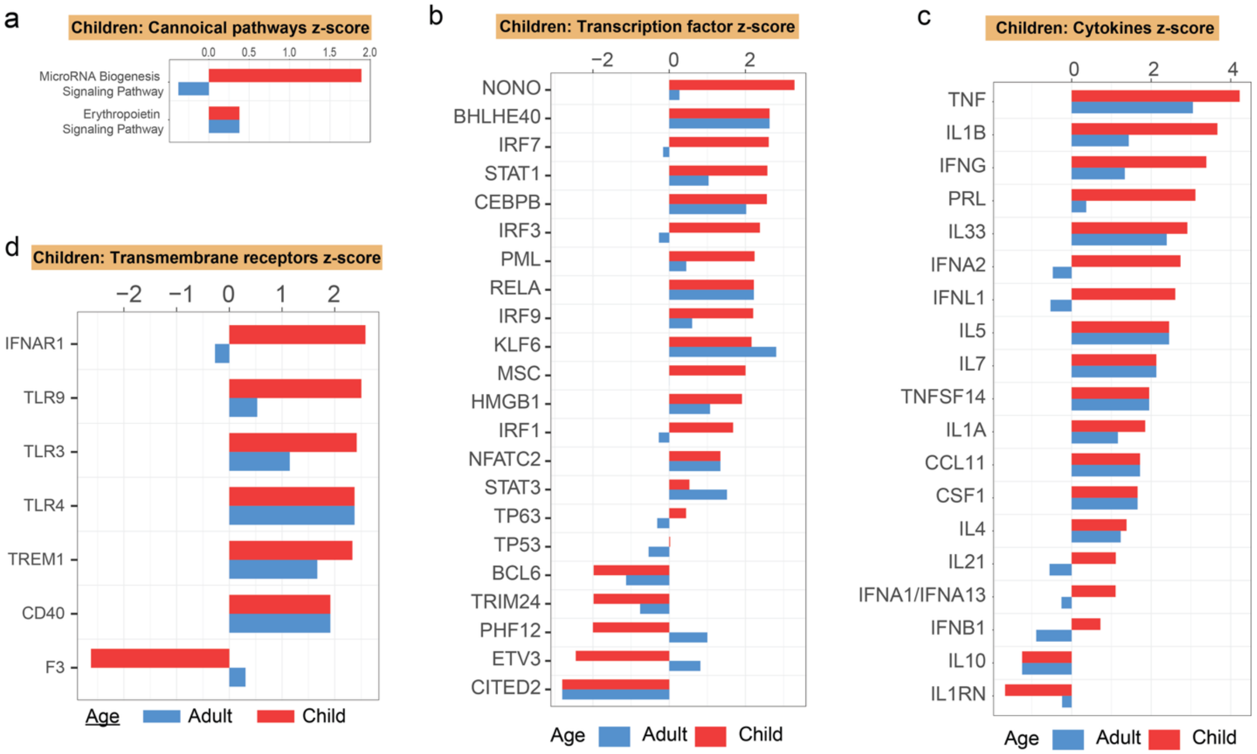

Supplementary Figure 7. IPA analysis of monocyte genes which were higher in children compared to adults after primary pRBC stimulation. **(a)** IPA performed using the log ratio value for children (red bars) or adults (blue bars) and the FDR q-value of the DEGs that were upregulated following stimulation and were higher in children (red bars, figure 3 (d)). Significant pathways depicted. Predicted activated or inhibited upstream regulators analysis in adults and children **(b)** transcription factors, **(c)** cytokines, **(d)** transmembrane receptors, using DEGs as in a. Benjamin-Hochberg corrected P-values used to identify significant pathways and upstream regulators in the IPA analysis. For all panels data is from children *n=5* and adults *n=5*.

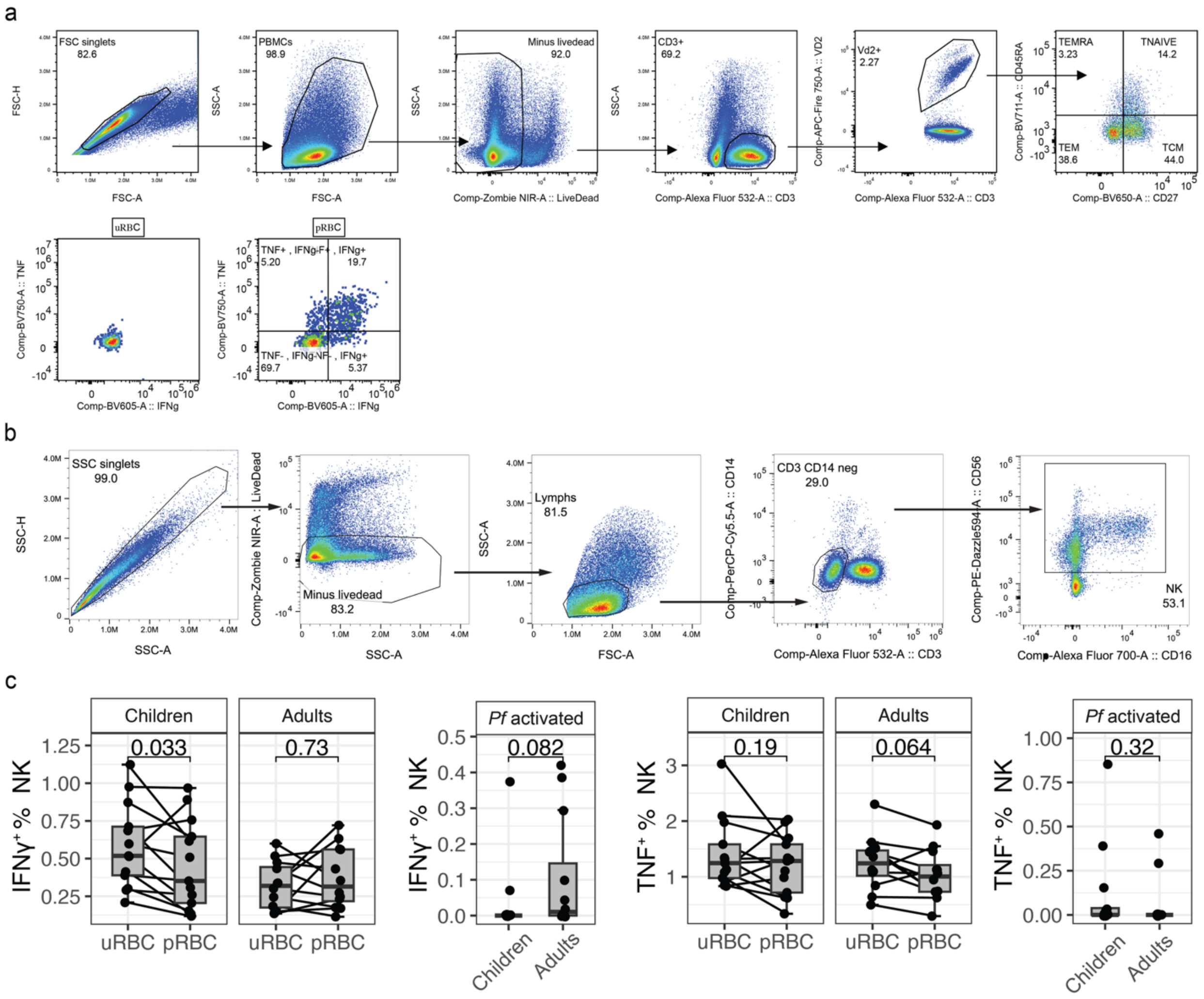

***Supplementary Figure 8. Representative gating strategy of Vδ2^{+} γδ T* cell, NK cells and NK cell cytokine capture.** PBMCs were stimulated with *P. falciparum*-infected (pRBCs) and uninfected (uRBCs) red blood cells with intracellular staining (children *n*=13, adults *n*=12). (**a**) Vδ2 γδ T cell gating strategy: Single+PBMC+Dead-CD3+Vδ2+ cells. Vδ2 γδ T cell memory subset defined by expression of CD27 and CD45RA (NAIVE: CD27^{+} CD45RA^{+}, CM: CD27^{+} CD45RA^{-}, EM: CD27^{-} CD45RA^{-} and EMRA: CD27^{-} CD45RA^{+}). Single- and co-producing IFNγ and TNF Vδ2 cells after uRBC and pRBC stimulation. (**b**) NK cell gating strategy: Single+Dead-Lymphocytes+CD3-CD14-CD56+ cells. (**c**) Intracellular cytokine capture of IFNγ and TNF by NK cells in children and adults during uRBC and pRBC stimulation. Proportion of *Pf* activated cytokine expressing NK cells (cytokine positive frequency in pRBC condition subtracted by uRBC condition). Lines represent paired observations. Wilcoxon signed rank test used for paired data. Mann-Whitney U test used for unpaired data. Centre line representing the median, box limits indicating the upper and lower quartiles, whiskers extending to 1.5 times the interquartile range. All p are two sided, with no adjustment for multiple comparisons. For all panels data is from children *n*=13 and adults *n*=12. *Pf* = *P. falciparum, p*RBC = parasitised red blood cells, uRBC = uninfected red blood cells.

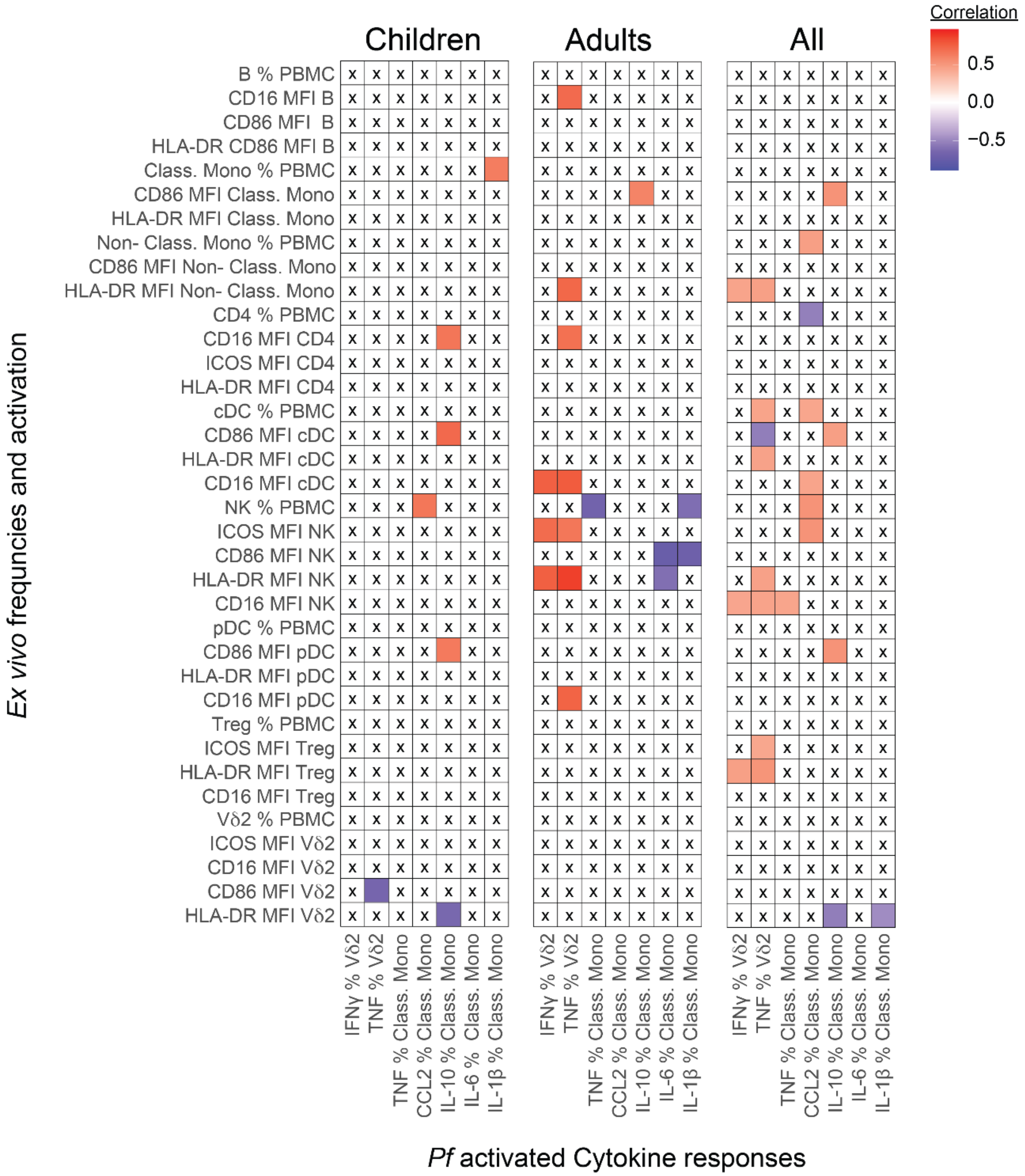

Supplementary Figure 9. Spearman's correlation matrix of ex vivo cell frequencies and activation with* P. falciparum *activated innate cell cytokine responses. *Ex vivo* cell frequencies and MFI of activation markers were correlated with *Pf* activated cytokine responses in classical monocytes and Vδ2 γδ T cells by Spearman's rank correlation coefficient in children, adult and all participants. Correlations with p>0.05 are blank/crossed and colour scales with Spearman's Rho. For all panels data is from children *n*=11 and adults *n*=11. MFI = median fluorescence intensity.

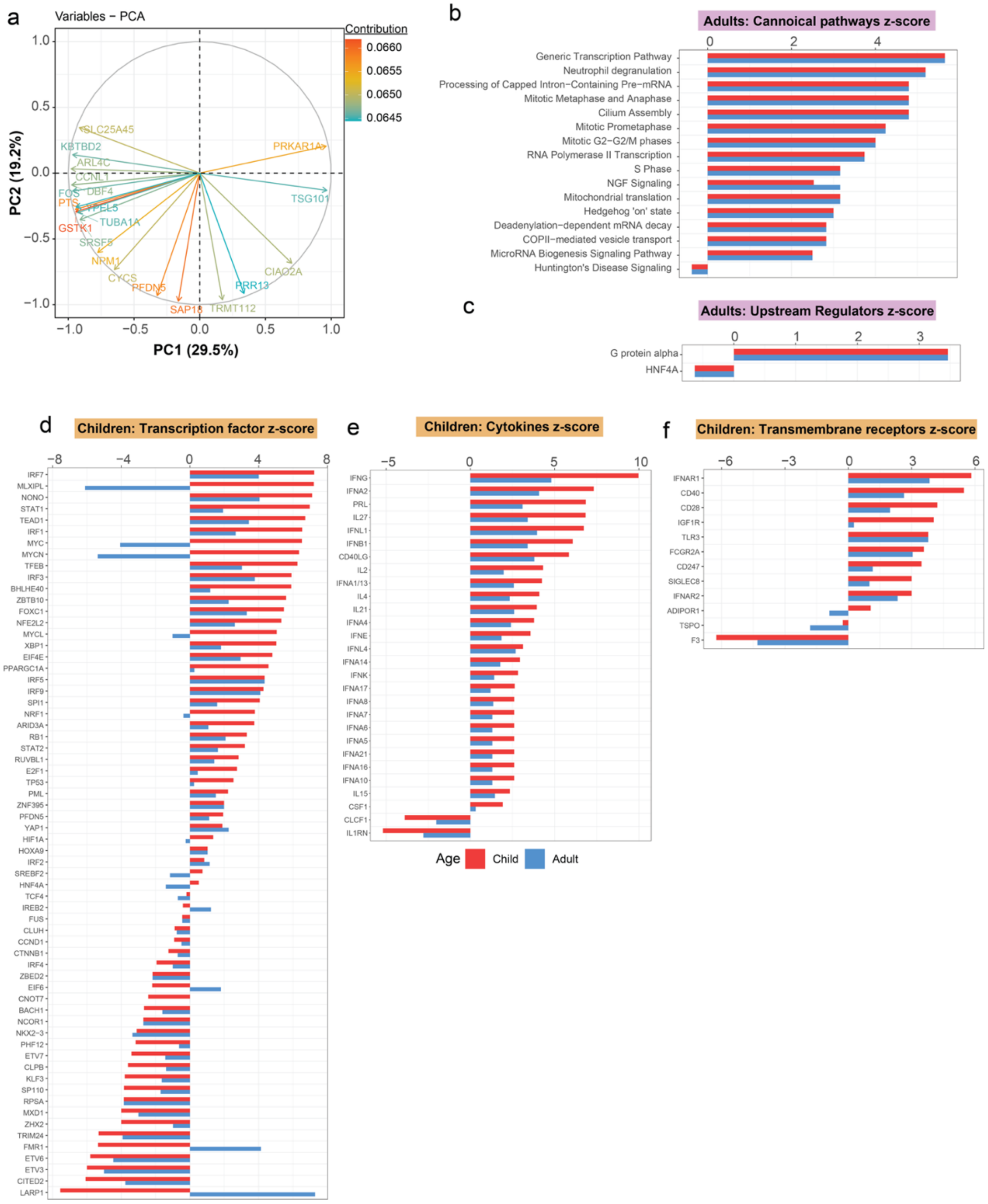

Supplementary Figure 10. IPA analysis of Vδ2 T cell genes which were higher in adults compared to children after primary pRBC stimulation. **(a)** Specific DEGs driving the variance for PCA1 and PCA2 in Vδ2 T cells. **(b)** IPA performed using the log ratio value for children (red bars) or adults (blue bars) and the FDR q-value of the DEGs that were upregulated following stimulation and were higher in adults (purple bars, Figure 5d). Significant pathways depicted. **(c)** Predicted activated or inhibited upstream regulators analysis in adults and children, using DEGs as in b. Benjamin-Hochberg corrected P-values

used to identify significant pathways and upstream regulators in the IPA analysis. Upstream regulator analysis for genes which were higher in children compared to adults after pRBC stimulation (orange bars, Figure 5d) showing predicted **(d)** transcription factors, **(e)** cytokines and **(f)** transmembrane receptors to be activated or inhibited in adults and children (purple bars, Figure 5d). For all panels data is from children *n=5* and adults *n=5*.

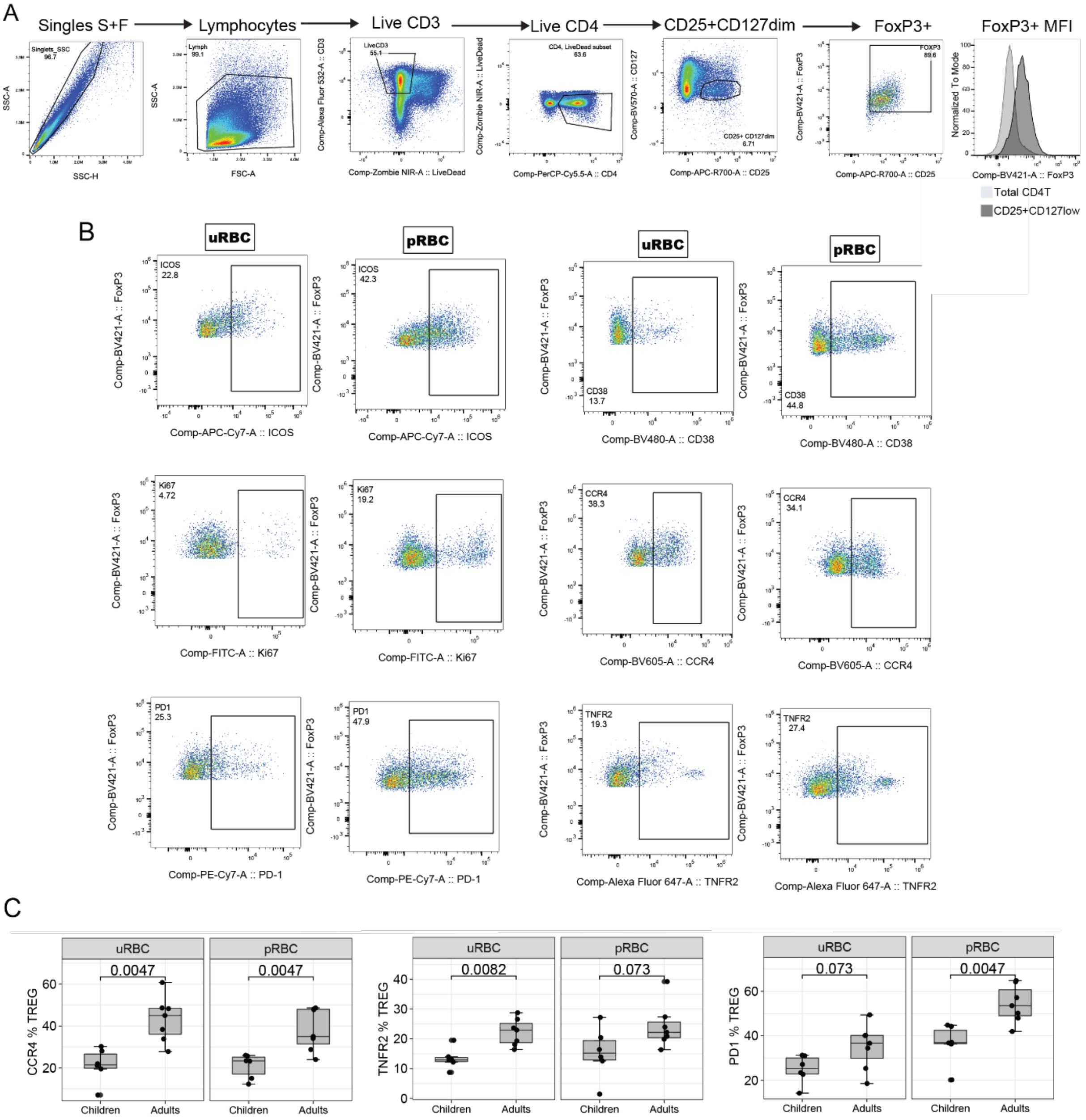

Supplementary Figure 11. Identification of regulatory T cells in malaria naive children and adults. (**a**) Full flow cytometry gating strategy to identify CD25high CD127low FoxP3high Tregs. (**b**) Flow cytometry gating examples for activation and inhibitory marker expression on Tregs after uRBC and pRBC stimulation. (**c**) uRBC and pRBC age comparisons for inhibitory markers (CCR4, TNFR2, PD1). Boxplots comparisons are Mann-Whitney U test. Centre line representing the median, box limits indicating the upper and lower quartiles, whiskers extending to the upper and lower limits. All p are two sided, with no adjustment for multiple comparisons. For all panels data is from children *n=6* and adults *n=7. p*RBC = parasitised red blood cells, uRBC = uninfected red blood cells, MFI = median fluorescence intensity.

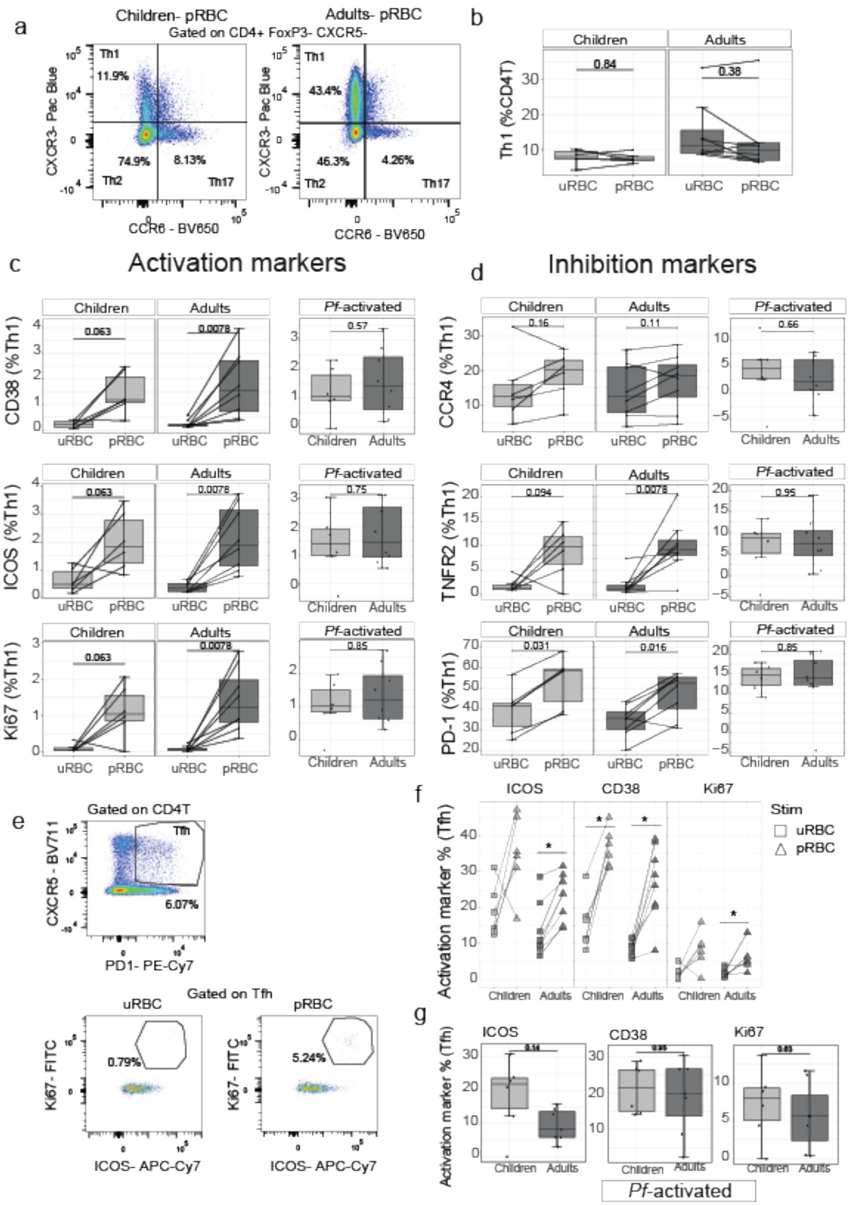

Supplementary Figure 12. Comparable Th1 CD4$^+$ T cells response in both adults and children in response to malaria. Th1 CD4$^+$ T cell surface marker expression in children (*n*=6) and adults (*n*=7) was measured after 5 days *in vitro* co-culture with trophozoite stage *P. falciparum* red blood cells (pRBCs) and uninfected red blood cells (uRBCs). (**a**) Th1 CD4$^+$ T cells were identified as CXCR3$^+$ CCR6$^-$ CD4 T cells (FoxP3$^-$CXCR5$^-$). **(b)** Th1 CD4$^+$ T cell frequency within CD4$^+$ T cells following stimulation. Surface marker frequency of inflammatory immune activation (**c**: CD38, ICOS and Ki67) and inhibition (**d**: CCR4, TNFR2 and PD-1) post stimulation of Th1 CD4$^+$ T cell frequency with pRBC, uRBC and *Pf* induced responses (quantified as expression in pRBC minus expression in uRBC cultured conditions). (**e**) Circulating Tfh were identified as CD4+ CXCR5+ PD-1+ cells, example plots of Tfh activation (Ki67 and ICOS) marker expression after uRBC or pRBC stimulation. (**f**) Frequency of inflammatory immune activation markers (ICOS, CD38 and Ki67) on Tfh following stimulation. (**g**) Age comparison of activation markers (ICOS, CD38 and Ki67) quantified as *Pf* activated (pRBC minus expression in uRBC cultured conditions). Lines represent paired observations. Wilcoxon signed rank test was used to compare paired data. Children and adult comparisons were made using the Mann-Whitney U test. Centre line representing

the median, box limits indicating the upper and lower quartiles, whiskers extending to the upper and lower limits. All p are two sided, with no adjustment for multiple comparisons. For all panels data is from children *n=6* and adults *n=7. Pf = P. falciparum, p*RBC = parasitised red blood cells, uRBC = uninfected red blood cells.

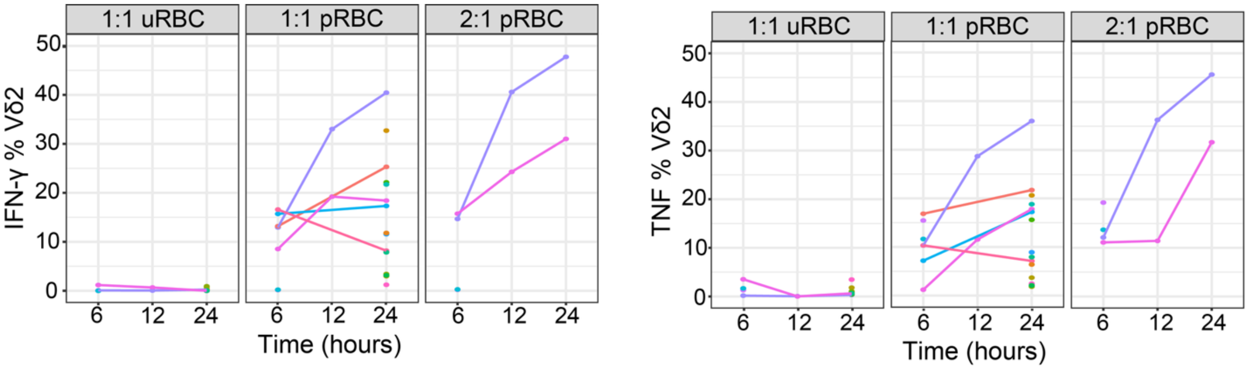

Supplementary Figure 13. Optimization of parasite ration and timing of in vitro innate stimulation. Intracellular cytokine capture of IFNγ and TNF in 1:1 uRBC (6hrs, *n* = 4; 12hrs, *n* = 2, 24 hrs, *n* = 11), 1:1 pRBC (6hrs, *n* = 7; 12hrs, *n* = 2, 24 hrs, *n* = 15) and 2:1 pRBC (6hrs, *n* = 4; 12hrs, *n* = 2, 24 hrs, *n* = 2). Colours represent individuals and lines are paired observations. *p*RBC = parasitised red blood cells, uRBC = uninfected red blood cells.
