# Supplementary material for: Age is an intrinsic driver of inflammatory responses to malaria
Source: Nat Commun. 2025 Sep 30;16:8665. doi: 10.1038/s41467-025-63638-1 (PMC12485045; doi:10.1038/s41467-025-63638-1)
Supplement: Supplementary file 2 — Description of Additional Supplementary Files [file 41467_2025_63638_MOESM2_ESM.pdf]

Description of Additional Supplementary Files

Supplementary Data 1: Classical monocyte gene expression variation between pre- and post-simulation in malaria-naïve children and adults. Gene expression data from FACS-isolated classical monocytes obtained from children and adults, both at baseline and following stimulation with *Plasmodium falciparum*–infected red blood cells, were analyzed using generalized linear mixed models implemented in the R package *glmmSeq*. Fold change due to stimulation was calculated by subtracting the log-transformed response term of unstimulated from stimulated samples. Fold change due to age was calculated by subtracting the log-transformed response term of children from adults for both stimulated and unstimulated samples. We corrected for multiple testing using Storey's q-value method, defining significance for q-values lower than 0.05 with the q-value R package. Code available at https://github.com/Boyle-Lab-CRDV/Age-is-an-intrinsic-driver-of-inflammatory-responses-to-malaria/.

Supplementary Data 2: Vδ2 T cell gene expression variation between pre- and post-simulation in malaria-naïve children and adults. Gene expression data from FACS-isolated Vδ2 T cells obtained from children and adults, both at baseline and following stimulation with *Plasmodium falciparum*–infected red blood cells, were analyzed using generalized linear mixed models implemented in the R package *glmmSeq*. Fold change due to stimulation was calculated by subtracting the log-transformed response term of unstimulated from stimulated samples. Fold change due to age was calculated by subtracting the log-transformed response term of children from adults for both stimulated and unstimulated samples. We corrected for multiple testing using Storey's q-value method, defining significance for q-values lower than 0.05 with the q-value R package. Code available at https://github.com/Boyle-Lab-CRDV/Age-is-an-intrinsic-driver-of-inflammatory-responses-to-malaria/.
