# Supplementary material for: Age is an intrinsic driver of inflammatory responses to malaria
Source: Nat Commun. 2025 Sep 30;16:8665. doi: 10.1038/s41467-025-63638-1 (PMC12485045; doi:10.1038/s41467-025-63638-1)
Supplement: Supplementary file 5 — Reporting Summary [file 41467_2025_63638_MOESM5_ESM.pdf]

nature portfolio

Corresponding author(s): Dr Michelle Boyle

Last updated by author(s): 14/07/25

Reporting Summary

Nature Portfolio wishes to improve the reproducibility of the work that we publish. This form provides structure for consistency and transparency in reporting. For further information on Nature Portfolio policies, see our Editorial Policies and the Editorial Policy Checklist.

Statistics

For all statistical analyses, confirm that the following items are present in the figure legend, table legend, main text, or Methods section.

n/a	Confirmed	
☐	☒	The exact sample size (*n*) for each experimental group/condition, given as a discrete number and unit of measurement
☐	☒	A statement on whether measurements were taken from distinct samples or whether the same sample was measured repeatedly
☐	☒	The statistical test(s) used AND whether they are one- or two-sided<br>*Only common tests should be described solely by name; describe more complex techniques in the Methods section.*
☐	☒	A description of all covariates tested
☐	☒	A description of any assumptions or corrections, such as tests of normality and adjustment for multiple comparisons
☐	☒	A full description of the statistical parameters including central tendency (e.g. means) or other basic estimates (e.g. regression coefficient) AND variation (e.g. standard deviation) or associated estimates of uncertainty (e.g. confidence intervals)
☐	☒	For null hypothesis testing, the test statistic (e.g. *F*, *t*, *r*) with confidence intervals, effect sizes, degrees of freedom and *P* value noted<br>*Give P values as exact values whenever suitable.*
☒	☐	For Bayesian analysis, information on the choice of priors and Markov chain Monte Carlo settings
☒	☐	For hierarchical and complex designs, identification of the appropriate level for tests and full reporting of outcomes
☐	☒	Estimates of effect sizes (e.g. Cohen's *d*, Pearson's *r*), indicating how they were calculated

Our web collection on statistics for biologists contains articles on many of the points above.

Software and code

Policy information about availability of computer code

Data collection	SpectroFlo v.2.2.0.3<br>Bioplex Manger v6.2<br>NextSeq v4.0.2
Data analysis	All analyses were performed using R v4.3.3 and RStudio v2023.12.1. R packages included: ggplot2 v3.5.2, dplyr v1.1.4, tidyr v1.3.1, tibble v3.2.1, purrr v1.0.4, stringr v1.5.1, reshape2 v1.4.4, readr v2.1.5, readxl v1.4.5, lubridate v1.9.4, forcats v1.0.0, cowplot v1.1.3, ggpubr v0.6.0, ggsignif v0.6.4, viridis v0.6.5, viridisLite v0.4.2, corrplot v0.95, factoextra v1.0.7, pheatmap v1.0.12, geomnet v0.3.1, visNetwork v2.1.2, igraph v2.1.4, Hmisc v5.2-3, glmmSeq v0.5.5, edgeR v3.40.2, limma v3.54.2, DESeq2 v1.38.3, BiocParallel v1.32.6, AnnotationDbi v1.60.2, org.Hs.eg.db v3.16.0, Biobase v2.58.0, SummarizedExperiment v1.28.0, S4Vectors v0.38.2, IRanges v2.34.1, GenomicRanges v1.50.2, GenomeInfoDb v1.34.9, MatrixGenerics v1.10.0, matrixStats v1.3.0, kableExtra v1.4.0, lme4 v1.1-37, car v3.1-3, carData v3.0-5, statmod v1.5.0, coda v0.19-4.1, ComplexHeatmap v2.15.4, Spectre v1.1.0, circlize v0.4.16, cluster v2.1.6, network v1.19.0, statnet.common v4.11.0, ggfortify v0.4.17, gridExtra v2.3, shiny v1.8.1.1, devtools v2.4.5, usethis v2.2.3, remotes v2.5.0, pkgbuild v1.4.4, pkgload v1.3.4, sessioninfo v1.2.2, profvis v0.3.8, rstatix v0.7.2, doParallel v1.0.17, foreach v1.5.2, and Spectre v1.1.0.<br><br>Additional software used includes: FlowJo v10, SpectroFlo v2.2.0.3, Cutadapt v1.9, STAR v2.5.2a, SAMtools v1.9, RSEM v1.2.30, RNA-SeQC v1.1.8, Ensembl v97 annotations, Ingenuity Pathway Analysis (IPA) v20.0, Python v3.6.1, and Perl v5.22.

For manuscripts utilizing custom algorithms or software that are central to the research but not yet described in published literature, software must be made available to editors and reviewers. We strongly encourage code deposition in a community repository (e.g. GitHub). See the Nature Portfolio guidelines for submitting code & software for further information.

Data

Policy information about availability of data

All manuscripts must include a data availability statement. This statement should provide the following information, where applicable:

- Accession codes, unique identifiers, or web links for publicly available datasets
- A description of any restrictions on data availability
- For clinical datasets or third party data, please ensure that the statement adheres to our policy

The datasets generated and analysed during the current study are publicly available in Gene Expression Omnibus and Flow Repository. The fastq files and the raw counts were deposit in the Gene Expression Omnibus (GEO) database under the accession number GSE270553 at: https://www.ncbi.nlm.nih.gov/geo/query/acc.cgi?acc=GSE270553.
FCS files are available at http://flowrepository.org/id/FR-FCM-Z8F2
Source Data are provided for this paper.

Research involving human participants, their data, or biological material

Policy information about studies with human participants or human data. See also policy information about sex, gender (identity/presentation), and sexual orientation and race, ethnicity and racism.

Reporting on sex and gender	Sex was assigned by enrolling study staff based on identity card/hospital records and information on gender was not collected. Some, male sex bias, due to a high proportion of infected males across all malaria species, possibly because of infection risk of forest worker in recruitment areas (https://doi.org/10.1093/cid/ciy065). Written informed consent was obtained from all adult study participant or, in the case of children, parents or legal guardians. No sex-based analysis have been performed due to the post-hoc nature of such analysis and due to limited sample sizes
Reporting on race, ethnicity, or other socially relevant groupings	n/a
Population characteristics	Age, sex, previous malaria (self-reported), CMV serostatus, allergy type, time since last allergic reaction.
Recruitment	Participants in the RDH cohort (malaria-naive) were recruited based on written informed consent for future immunological studies. Samples selected for this study were selected on not having experienced an allergic reaction in the past 2 months and a balanced age distribution between groups. Children wer defined as <12 and adults >18 years old.<br><br>For Malaysian hospital sites patients presenting to study hospitals with microscopy-diagnosed malaria of any species were enrolled following written informed consent (further info: https://doi.org/10.1093/cid/cis902, https://doi.org/10.1093/cid/ciy065). Children were defined as age <12 years, based on Malaysian Ministry of Health pediatric ward admission guidelines.
Ethics oversight	Written informed consent was obtained from all study participants or, in the case of children, parents or guardians. Studies were approved by the ethics committees of the Northern Territory Department of Health and Menzies School of Health Research (Darwin, Australia, HREC 2010-1431, HREC-2012-1766), Medical Research and Ethics Committee, Ministry of Health, Malaysia (NMRR-10-754-6684 and NMRR-12-499-1203), QIMR-Berghofer Human Research Ethics Committee (HREC P3445 and P3444) and the Alfred Hospital Ethics Committee (HREC 188/23 and 80/24).

Note that full information on the approval of the study protocol must also be provided in the manuscript.

Field-specific reporting

Please select the one below that is the best fit for your research. If you are not sure, read the appropriate sections before making your selection.

☒ Life sciences ☐ Behavioural & social sciences ☐ Ecological, evolutionary & environmental sciences

For a reference copy of the document with all sections, see nature.com/documents/nr-reporting-summary-flat.pdf

Life sciences study design

All studies must disclose on these points even when the disclosure is negative.

Sample size	Sample size was restricted by availability. For Bulk RNAseq experiments, we used 5 children and 5 adults from the malaria-naive cohort (n=10), for flow cytometry experiments we used 13 children and 13 adults from the malaria-naive cohort, across the different panels performed (n=26). For analysis of plasma analytes, all individuals from the clinical Sabah cohort with available plasma were included (n=76).
Data exclusions	For RNAseq data and flow cytometry data there were no exclusions. For multiplex assays wells with a bead count of ≤50 were excluded from analysis.
Replication	For flow cytometry experiments, all panel optimization was performed on healthly malaria naive samples prior to analysis in this study. Panel optimization was check for reproducibility. For application to study samples, flow cytometry experiments were performed once due to limited cell numbers and patient samples.

Randomization	n/a
Blinding	Blinding is not relevant in study recruitment as patients were presented to hospital with malaria for enrollment. This current study was not linked to parent study clinical trials

Reporting for specific materials, systems and methods

We require information from authors about some types of materials, experimental systems and methods used in many studies. Here, indicate whether each material, system or method listed is relevant to your study. If you are not sure if a list item applies to your research, read the appropriate section before selecting a response.

Materials & experimental systems

n/a	Involved in the study
☐	☒ Antibodies
☒	☐ Eukaryotic cell lines
☒	☐ Palaeontology and archaeology
☒	☐ Animals and other organisms
☒	☐ Clinical data
☒	☐ Dual use research of concern
☒	☐ Plants

Methods

n/a	Involved in the study
☒	☐ ChIP-seq
☐	☒ Flow cytometry
☒	☐ MRI-based neuroimaging

Antibodies

Antibodies used

Antigen	Fluorochrome	Clone	Manufacturer	Cat #	Dilution
CXCR3	BV421	1C6	BD Biosciences	562558	1/50
CD86	BV480	2331	BD Biosciences	566131	1/100
CD14	BV510	M5E2	Biolegend	301842	1/50
CD127	BV570	A019D5	Biolegend	351308	1/100
CCR6	BV650	11A9	BD Biosciences	563922	1/100
CXCR5	BV711	J252D4	Biolegend	356934	1/50
HLA-DR	BV785	L243	Biolegend	307642	1/50
HLADR	BV570	L243	Biolegend	307637	1/50
CD45RA	BB515	HI100	BD Biosciences	564552	1/1000
CD3	FITC	SK7	Biolegend	344804	1/10
CD4	PerCPCy5.5	OKT4	Biolegend	317428	1/400
CD19	PE	HIB19	Biolegend	302208	1/10
CD56	PE-dazzle	HCD56	Biolegend	318347	1/100
PD-1	PE-CY7	EH12.1	BD Biosciences	561272	1/100
VD2	APC	B6	Biolegend	331418	1/500
FoxP3	AF647	206D	Biolegend	320114	1/25
CD16	AF700	3G8	Biolegend	302026	1/2500
ICOS	APC-Cy7	C398.4A	Biolegend	301820	1/100
Viability	NIR		Invitrogen	L34975	1/1500
IL-12	BV421	C8.6	BD Biosciences	565023	1/33
IFNg	BV605	B27	BD Biosciences	562974	1/25
CD27	BV650	O325	Biolegend	302827	1/400
CD45RA	BV711	H100	Biolegend	304137	1/1000
TNF	BV750	Mab11	BD Biosciences	566359	1/100
CD3	AF352	UCHT1	Invitrogen	58003842	1/50
IL-1b	FITC	CRM56	Invitrogen	11-7018-42	1/100
CD14	PerCPCy5.5	M5E2	Biolegend	301823	1/400
IL-10	PE	JES3-9D7	BD Biosciences	559337	1/10
IL-6	PE-Cy7	MQ2-13A5	Biolegend	501119	1/100
Granzyme B	APC	QA16A02	Biolegend	372203	1/1000
MCP-1	AF647	5D3-F7	BD Biosciences	563496	1/150
Vd2	APC-FIRE	B6	Biolegend	331419	1/500
FOXP3	BV421	206D	Biolegend	320124	1/25
CXCR3	PAC Blue	G025H7	Biolegend	353723	1/50
CCR4	BV605	L291H4	Biolegend	359417	1/100
CCR7	BV786	3D12	BD Biosciences	563710	1/50
Ki67	FITC	B56	BD Biosciences	556026	1/100
TNFR2	AF647	hTNFR-M1	BD Biosciences	562909	1/50
CD25	AF700	2A3	BD Biosciences	565106	1/250

| Viability | Sytox Blue | - | Invitrogen | S11348 | 1/200
| CD56 | BV510 | HCD56 | Biolegend | 318340 | 1/50
| CD19 | BV605 | SJ25C1 | BD Biosciences | 562653 | 1/50
| CD3 | BV650 | OKT3 | Biolegend | 317324 | 1/50
| CD14 | PE | M5E2 | BD | 561707 | 1/50
| CD64 | PE-DAZZLE 594 | 10.1 | Biolegend | 305032 | 1/100

Validation

Antibodies were purified by affinity chromatography and conjugated under optimal conditions by the manufacturer. Validation was performed by manufacturer. Each antibody lot is FC quality control tested by immunofluorescent staining with flow cytometric analysis. Monoclonal antibodies have verified reactivity with human species and are for Research Use Only (RUO)

Plants

Seed stocks

n/a

Novel plant genotypes

n/a

Authentication

n/a

Flow Cytometry

Plots

Confirm that:

- [x] The axis labels state the marker and fluorochrome used (e.g. CD4-FITC).
- [x] The axis scales are clearly visible. Include numbers along axes only for bottom left plot of group (a 'group' is an analysis of identical markers).
- [x] All plots are contour plots with outliers or pseudocolor plots.
- [x] A numerical value for number of cells or percentage (with statistics) is provided.

Methodology

Sample preparation

PBMCs were isolated from whole blood via density centrifugation with Ficoll-Paque prior to cryopreservation. Thawed peripheral blood mononuclear cells were stained ex vivo or after in vitro stimulation. Cells were stained Viability dye, washed twice in 2% FCS/PBS, surface stained with listed antibodies then washed twice in 2% FCS/PBS. In vitro stimulated cells were fixed/permeablised with 1X BD Cytofix/Cytoperm™ Fixation then washed twice with 1X BD Permeablization Buffer. Cells were respended in 2% FCS/PBS for sample acquisition.

Instrument

Cytek® Aurora 3-Laser Spectral Flow Cytometer (R0021)

Software

FlowJo v10, SpectroFlo v2.2.0.3, R v4.3.3, RStudio v2023.12.1. and Spectre v1.1.0.

Cell population abundance

Post-sort purity was determined by re-acquiring a combined aliquot of all cell type isolations and determining purity as cell subset % total cells.
Classical Monocytes: 95.7%
Vδ2 T cells: 92.5%

Gating strategy

Ex vivo cell phenotyping comparison of RDH samples: Doublets removed and PBMCs gated by granularity and size with FSC-A .v SSC-A and live cells were Viability NIR-. Cell subsets were identified by UMAP clustering and expression of lineage markers: Tregs (CD3+CD4+FOXP3+CD127dimCXCR5-CD19-), CD4 T cells (CD3+CD4+FOXP3-CD127highCXCR5-CD19-), Tfh(CD3+CD4+FOXP3-CD127highCXCR5+CD19-), Vδ2 T cell (CD3+Vδ2+CD19-), CD3+CD4- T cells(CD3+CD4-CD19-), B cells(CD19+HLA-DR+), NK cells(CD56+CD16+CD3-), Plasmacytoid DCs(HLA-DR+CD3-CD19-CD14-CD56-CD16-), CD14+ monocytes(CD3-CD19-CD56-CD14+HLA-DR+CD86+CD16-), classical DCs(CD3-CD19-CD56-CD14-HLA-DR+CD86+CD16-),CD16+ monocytes(CD3-CD19-CD56-CD14+HLA-DR+CD86+CD16+) and lineage negative cells.

In vitro stimulation of RDH samples: Doublets removed and PBMCs gated by granularity and size with FSC-A .v SSC-A and live cells were Viability NIR-. Cells subsets were then gated:
Classical Monocytes: Monocytes gated granularity and size with FSC-A v SSC-A, CD3-HLA-DR+CD86+CD14+, IL-10+, IL-6+, IL-1β+, TNF+, CCL2+.
Non-classical Monocytes: Monocytes gated granularity and size with FSC-A v SSC-A, CD3-HLA-DR+CD86+CD14-, IL-10+, IL-6+, IL-1β+, TNF+, CCL2+.
Vδ2 T cells: CD3+Vδ2+, CD27+/-CD45RA+/- (Memory subsets), IFNγ+, TNF+
NK cells: CD3-CD14-CD56+, IFNγ+, TNF+

Tregs: CD3+CD4+CD25+CD127dimFoxP3+, ICOS+, CD38+, Ki67+, CCR4+, PD-1+, TNFR2+.

FACS Sort of RDH samples for RNA-seq:
Vδ2 T cells: Monocytes gated granularity and size with FSC-A v SSC-A, Sytox Blue-CD56-CD3+Vδ2+
Classical Monocytes: Lymphocytes gated granularity and size with FSC-A v SSC-A, Sytox Blue-CD56-CD3+HLA-DR+CD64+

☒ Tick this box to confirm that a figure exemplifying the gating strategy is provided in the Supplementary Information.
